# Immunomodulatory Activity of a Novel, Synthetic Beta-glucan (β-glu6) in Murine Macrophages and Human Peripheral Blood Mononuclear Cells

**DOI:** 10.1371/journal.pone.0080399

**Published:** 2013-11-06

**Authors:** Xiaofei Li, Jing Wang, Wei Wang, Chunhong Liu, Shuhui Sun, Jianxin Gu, Xun Wang, Diana Boraschi, Yuxian Huang, Di Qu

**Affiliations:** 1 Key Laboratory of Medical Molecular Virology of MOE and MOH, Institute of Medical Microbiology and Institutes of Biomedical Sciences, Shanghai Medical College of Fudan University, Shanghai, China; 2 Shanghai Municipal Center for Disease Control and Prevention, Shanghai, China; 3 Key Laboratory of Glycoconjugates Research Ministry of Public Health, Shanghai Medical College of Fudan University, Shanghai, China; 4 Shanghai Blood Center, Shanghai, China; 5 Laboratory of Cytokines, Unit of Immunobiology, Institute of Biomedical Technologies, National Research Council, Pisa, Italy; 6 Department of Infectious Diseases, Huashan Hospital, Shanghai Medical College of Fudan University, Shanghai, China; Istituto Superiore di Sanità, Italy

## Abstract

Natural β-glucans extracted from plants and fungi have been used in clinical therapies since the late 20th century. However, the heterogeneity of natural β-glucans limits their clinical applicability. We have synthesized β-glu6, which is an analog of the lentinan basic unit, β-(1→6)-branched β-(1→3) glucohexaose, that contains an α-(1→3)-linked bond. We have demonstrated the stimulatory effect of this molecule on the immune response, but the mechanisms by which β-glu6 activates innate immunity have not been elucidated. In this study, murine macrophages and human PBMCs were used to evaluate the immunomodulatory effects of β-glu6. We showed that β-glu6 activated ERK and c-Raf phosphorylation but suppressed the AKT signaling pathway in murine macrophages. Additionally, β-glu6 enhanced the secretion of large levels of cytokines and chemokines, including CD54, IL-1α, IL-1β, IL-16, IL-17, IL-23, IFN-γ, CCL1, CCL3, CCL4, CCL12, CXCL10, tissue inhibitor of metalloproteinase-1 (TIMP-1) and G-CSF in murine macrophages as well as IL-6, CCL2, CCL3, CCL5, CXCL1 and macrophage migration inhibitory factor (MIF) in human PBMCs. In summary, it demonstrates the immunomodulatory activity of β-glu6 in innate immunity.

## Introduction

Beta-glucans derived from yeast and medicinal mushrooms are potent immunomodulators of both innate and adaptive immunity. Beta-glucans are heterogeneous polysaccharides composed of glucose polymers that exhibit variable activities due to different molecular weights, structures, frequencies of branching and solubility. The basic unit in β-glucans, β-(1→6)-branched β-(1→3) glucohexaose, is reported to play a major role in anti-tumor activity [1], and its stimulatory effects are similar to lentinan [2]. Many receptors that recognize β-glucans have been described. Brown et al. showed that Dectin-1 was a pattern-recognition receptor (PRR) that recognized a variety of glucans from fungi and plants [3]. Jouault et al. reported that TLR2 was required for *C. albicans* uptake and endocytosis [4], and Thornton’s group reported that the soluble zymosan polysaccharide (SZP) had a high affinity for CR3 [5]. Nevertheless, identifying and characterizing the receptors of natural β-glucans is problematic, and consequently, developing new, single-entity drugs is challenging because of the considerable variation in the structure of β-glucans. 

In this study, we have used a new synthetic β-glucan, a β-(1→6)-branched β-(1→3) glucohexaose analog (referred to as β-glu6 in this paper), which contains six glucoses with an α-(1→3)-linked bond (β-D-Glcp-(1→3)-[β-D-Glcp-(1→6)-] β-D-Glcp-(1→3)-α-D-Glcp-(1→3)-[β-D-Glcp-(1→6)-]D-Glcp) [1]. This molecule promoted the maturation of macrophages and DCs and greatly enhanced the titer of HBsAg-specific antibodies in BALB/c mice [6]. Moreover, β-glu6 has been reported to enhance the virus-specific Th1 response induced by the pB144 plasmid, which was constructed by inserting a gene fragment encoding the N terminal 144 amino acids of HBcAg into pcDNA3.1 under the control of the CMV immediate-early promoter [7]. However, the mechanisms by which β-glucan stimulates the immune response have not been elucidated, especially in innate immune cells. 

Compared with other immune cells, macrophages are long lived and produce high levels of cytokines and chemokines upon stimulation to recruit immune cells to the site of infection [8]. After activation, macrophages differentiate into two main subpopulations depending on the cytokine environment: classically activated macrophages (M1s) and alternatively activated macrophages (M2s) [9,10]. M1s are induced by IFN-γ plus TNF-α or TLR ligands, and they secrete inflammatory cytokines, including IL-6, IL-12, TNF-α, IL-1β and IL-23. After exposure to IL-4 and IL-13, M2s secrete the anti-inflammatory cytokines IL-1Ra, IL-10 and TGF-β, which provide immunosuppressive and healing effects [11]. 

Many signaling pathways are involved in cell activation, differentiation and cytokine secretion of macrophages. The Ras-Raf-MEK-ERK and PI3K-Akt signaling pathways in macrophages are the most commonly studied intracellular transduction cascades. In the Ras-Raf-MEK-ERK pathway, activated Ras (a single-subunit small GTPase) activates RAF kinase, which phosphorylates and directly leads to MEK (MEK1 and MEK2) activation; then, MEK phosphorylates and activates ERK [12]. ERK plays a key role in activating oxidative and nitrosative bursts, polarizing macrophages, and programming gene expression in the nucleus [13,14]. The PI3K/Akt pathway also plays a central role in diverse cellular processes, including cell survival, proliferation and differentiation [15,16]. The phosphatase PTEN (phosphatase and tensin homolog) dephosphorylates and thus terminates the activity of PIP3, which is generated by PI3K and recruits target proteins such as Phosphoinositide-dependent kinase-1 (PDK-1) to the membrane [17]. Akt, a master kinase for IκB kinase, glycogen synthase kinase 3 (GSK-3) and other substrates, can then be phosphorylated by PDK-1 at threonine 308 and mTORC2 at serine 473 to control downstream events [18] such as the expression of cytokine genes. 

In this study, we found that β-glu6 activated ERK and c-Raf phosphorylation but suppressed the AKT signaling pathway. Antibodies against TLR2, TLR4 and Dectin-1 inhibited the β-glu6-induced alterations in the Ras-Raf-MEK-ERK and PI3K-Akt signaling pathways in murine macrophages. Moreover, we examined the cytokine and chemokine profiles in β-glu6-stimulated murine macrophages and human PBMCs and proved the immunomodulatory activity of β-glu6 in innate immunity. 

## Materials and Methods

### Synthetic β-glucan

Beta-glu6 was synthesized by Fanzuo Kong, Jun Ning and Jianxin Gu. The details of synthesis are described in a United States Patent (patent number: US7, 365, 191, B2) and a Chinese Patent (patent number: ZL02813563.6). The structure of β-glu6 was identified with NMR, MS and HPLC. The purity of β-glu6 is more than 98% [6]. Beta -glu6 was dissolved in sterile phosphate-buffered saline (PBS) at a concentration of 100 mg/mL as a stock solution, and endotoxin contamination in β-glu6 was assessed by the Limulus amebocyte lysate (LAL) colorimetric assay (Lonza, Walkersville, MD, USA). 

### Mice

Female inbred C57BL/6 mice (aged 6–8 weeks) were purchased from the Animal Center of Chinese Academy of Sciences (Shanghai, China) and maintained in pathogen-free housing. All animal procedures were performed in compliance with the criteria outlined in the Guide for the Care and Use of Laboratory Animals (prepared by the National Academy of Sciences and published by the National Institutes of Health) and were approved by the Institutional Animal Care and Use Committee (IACUC) of Shanghai Medical College of Fudan University (IACUC Animal Project Number: 20100913). All surgery was performed under sodium pentobarbital anesthesia, and all efforts were made to minimize suffering. 

### Reagents

LPS (from *E. coli* strain 055:B5) was purchased from Sigma-Aldrich. Brewer thioglycollate medium was purchased from Kang Run Biology Science (Shanghai, China). Monoclonal anti-mouse antibodies against phospho-ERK, ERK and β-actin as well as the phospho-Akt pathway antibody sampler kit were purchased from Cell Signal Technology (Beverly, MA, USA). Anti-TLR2 (clone mT2.7) and anti-TLR4 (clone UT41) monoclonal antibodies were purchased from eBioscience (San Diego, CA, USA), and the anti-Dectin-1 monoclonal antibody (clone 2A11) was purchased from Hycult Biotech (PB Uden, The Netherlands). 

### Cell culture and treatment

Peritoneal macrophages of female C57BL/6 mice were induced by intraperitoneal injection of 3% Brewer thioglycollate medium (1.5 mL) 5 days prior to cell harvest and were obtained by washing the peritoneal cavity. Cells were resuspended in pre-warmed RPMI-1640 medium containing 10% FBS, 100 mg/L streptomycin and 10^5^ U/L penicillin (GIBCO Invitrogen, Grant Island, NY, USA) and distributed at 5×10^6^ cells/well in 6-well BD Falcon™ culture plates (BD Biosciences, San Jose, CA, USA). Macrophages were allowed to adhere for 2 h at 37°C in a humidified atmosphere containing 5% CO_2_, and non-adherent cells were removed by washing the plates twice with PBS. The resulting adherent population consisted of 95% peritoneal macrophages. Pooled murine macrophages were pre-treated with or without PD98059 (50 μM), anti-TLR2, anti-TLR-4 or anti-Dectin-1 antibodies (10 μg/mL) for 1 h. Then, β-glu6 (100 μg/mL) was added to the peritoneal macrophages and cultured for an appropriate time to detect protein phosphorylation, cytokine gene transcription and cytokine secretion.

Freshly drawn heparin-anticoagulated human blood from three healthy donors were kindly provided by Shanghai Blood Center. After a Ficoll gradient centrifugation (Pharmacia, Uppsala, Sweden), PBMCs were collected and washed in RPMI -1640 medium (Life Technologies) supplemented with 1% (v/v) fetal bovine serum (FBS; Life Technologies). Freshly isolated PBMCs (5×10^6^ cells/well) were seeded in 6-well plates in RPMI-1640 medium containing 10% FBS, 100 mg/L streptomycin and 10^5^ U/L penicillin. After incubation with or without β-glu6 (100ug/mL) for 24 h, the cell culture supernatants were collected for proteome profiler human cytokine assay.

### MTT assay

Macrophage viability was assessed by the mitochondria-dependent reduction of MTT to formazan (Amresco, Solon, Ohio, USA). In 96-well plates, macrophages (2×10^4^ cells/well) were treated with β-glu6 at different concentrations (0-1000 μg/mL) or LPS (1 μg/mL) for 48 h. The MTT solution was added at a final concentration of 0.5 g/L, and the cells were further incubated for 4 h at 37°C with 5% CO_2_. The culture medium was discarded, and 200 μL of DMSO was added to resuspend the formazan crystals. The absorbance at 490 nm was measured by DTX Series Multimode Detectors (Beckman Coulter, California, USA).

### RNA isolation and real-time RT-PCR

Peritoneal macrophages were treated with β-glu6 for 4 h or 24 h. Total RNA was extracted using TRIzol reagent (Invitrogen) and quantified by UV spectroscopy using a biophotometer, followed by DNase digestion, as recommended by the manufacturer. The integrity and quality of isolated RNA were determined by agarose gel electrophoresis. Reverse transcription was performed with the Reverse Transcription System (Promega) using 2 μg of RNA. Real-time PCR was performed on an Applied Biosystems 7500 Real-Time PCR System (Life Technologies) using 10 μL of 2×SYBR^®^ Premix Ex Taq Mixes (Takara), 0.4 μL of sense and anti-sense primers (10 μM), 7.2 μL of DNase/RNase-Free water and 2 μL of diluted cDNA samples, for a total of 20 μL per well. The cycling conditions were as follows: 30 s at 95 °C and then 40 cycles of 5 s at 95 °C and 30 s at 60 °C. The relative expression levels for cytokine genes were determined using the comparative Ct (2^-ΔΔCt^) method according to the ABI Prism 7700 User Bulletin 2 (Life Technologies), and the GAPDH gene was used as an internal reference. 

Primers used in this study are listed in Table 1.

**Table 1:** Primer sequences for murine cytokines.

**Mouse cytokines**	**Accession number**	**5'-3' primer sequence**
TNF-α	NM_013693	F: GTGGAACTGGCAGAAGAG
		R: CCATAGAACTGATGAGAGG
IL-1β	NM_008361	F: TGGGAAACAACAGTGGTCAG
		R: CCATCAGAGGCAAGGAGGA
IL-1Ra	NM_031167	F: ACAGTAGAAGGAGACAGAAG
		R: GGTGGTAGAGCAGAAGAC
IL-6	NM_031168	F: TGCCTTCTTGGGACTGATG
		R: ACTCTGGCTTTGTCTTTCTTGT
IL-10	NM_010548	F: GAAGACCCTCAGGATGCG
		R: CCAAGGAGTTGTTTCCGTTA
IL-12p40	NM_008352	F: AGATGAAGGAGACAGAGGAG
		R: GCACGAGGAATTGTAATAGC
GAPDH	NM_008084	F: GGTGAAGGTCGGTGTGAACG
		R: CTCGCTCCTGGAAGATGGTG
IFN-γ	NM_008337	F: ATGAACGCTACACACTGCATC
		R: CCATCCTTTTGCCAGTTCCTC
IL-4	NM_021283	F: GGTCTCAACCCCCAGCTAGT
		R: GCCGATGATCTCTCTCAAGTGAT

F: forward primer; R: reverse primer

Sequences for primers were obtained from Genbank and NCBI. Primers were designed to span intron-exon boundaries to prevent amplification of possible genomic DNA using Beacon Designer (Palo Alto, CA, USA), and BLAST searches were performed to ensure specificity. Finally, the primers were synthesized at Sangon Biotech (Shanghai, China).

### Proteome Profiler Mouse Cytokine Array

The Proteome Profiler™ Array (Mouse Cytokine Array Panel A & Human Cytokine Array Panel A) from R&D Systems (Minneapolis, MN, USA) was used to detect the relative levels of cytokines and chemokines secreted by mouse peritoneal macrophages and human PBMCs treated with β-glu6. Briefly, 5×10^6^ cells/well in 6-well plates were treated with β-glu6 for 24 h, and the cell culture supernatants were collected. The samples were mixed with 15 μL of the reconstituted detection antibody cocktail and incubated for 1 h at room temperature. Then, the sample/antibody mixtures were added to the membranes, which had already been blocked with Array Buffer 1 and incubated overnight at 4°C on a rocking platform. After three washes (5 min/wash) with TBST, the membranes were incubated in diluted streptavidin-HRP for 30 minutes at room temperature. After washing the membranes, dots on the membranes were detected by an enhanced chemiluminescence kit (Thermo scientific, MA, USA), as directed by the manufacturer. Membranes were then exposed to X-ray film for 1 or 5 minutes, and a densitometric analysis of the intensities of the cytokine dots was performed with Quantity One software (Bio-Rad).

### Preparation of cell extracts and Western Blotting

 Macrophages (5×10^6^ cells/well) were treated with β-glu6 for 2 h, 4 h or 6 h. After being washed with PBS, the cells were lysed in lysis buffer (20 mmol/L Tris pH 7.5, 150 mmol/L NaCl, 1 mmol/L Na_2_EDTA, 2.5 mmol/L sodium pyrophosphate, 1% Triton X-100, 1 mmol/L β-glycerophosphate, 1 mmol/L Na_3_VO4, 1 μg/mL Leupeptin and 1 mmol/L PMSF) on ice and then centrifuged (14000 g, 5 min, 4 °C). The supernatants of the cell lysates were mixed with 5× protein loading buffer, boiled for 5 min at 100 °C, analyzed by 12% SDS-PAGE, transferred to PVDF membrane (Millipore, MA, USA) and blocked with 5% nonfat milk in TBST for 2 h at room temperature. The membranes were probed with specific primary antibodies (phosphorylated ERK 1/2, total ERK 1/2, Akt (Thr308), phosphorylated PTEN, PDK-1, GSK-3β, and β-actin) overnight at 4°C. After being washed, the membranes were incubated with the corresponding anti-rabbit IgG-HRP conjugated antibody (diluted at 1:2,000 in TBST with 5% milk) for 1 h at room temperature, and the protein bands were detected by a chemiluminescence kit. The membranes were exposed to X-ray film, and a densitometric analysis of the intensities of the protein bands was performed with Quantity One software.

### Statistical analysis

The experimental data were analyzed using SPSS statistical software (version 16.0) (SPSS Inc., Chicago, IL, USA) and presented as the mean ± SD from at least three independent experiments. Differences between groups were analyzed by an ANOVA; means of groups were compared by a t-test. P < 0.05 was considered statistically significant.

## Results

### β-glu6 (G) is non-toxic and suppresses AKT phosphorylation in macrophages

**Figure 1:**
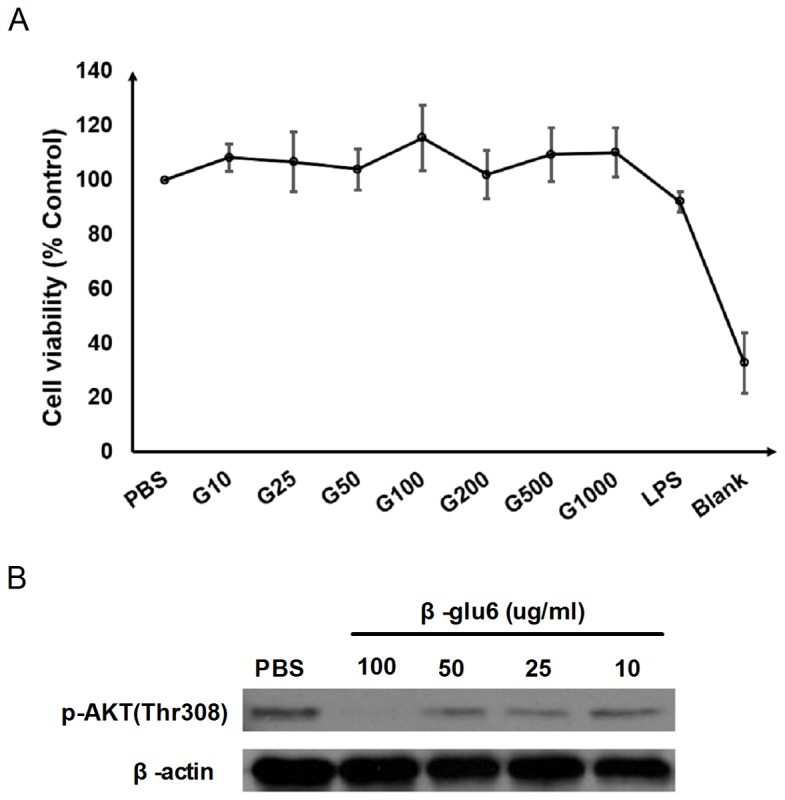
β-glu6 (G) suppresses AKT phosphorylation and does not affect peritoneal macrophage viability. The peritoneal macrophages were treated with 10 μg/mL, 25 μg/mL, 50 μg/mL, 100 μg/mL, 200 μg/mL, 500 μg/mL and 1000 μg/mL of β-glu6 (G) or 1 μg/mL of LPS, and the viability of the macrophages was examined by the MTT assay, as described in the Materials and Methods section. Data are expressed as the mean ± SD of three independent experiments (A). Macrophages were treated with 10 μg/mL, 25 μg/mL, 50 μg/mL, and 100 μg/mL of β-glu6 (G) for 2 h, and the level of phosphorylated AKT (Thr308) was detected by western blot assay. Beta-actin was used as a loading control. Data shown represent one of three independent experiments (B).

The purity of β-glu6 was more than 98% as determined by HPLC [6]. The endotoxin in β-glu6 was under the detection limit (0.1 EU/mL) by a quantitative chromogenic LAL assay, as shown in Table S1. No significant difference in cell viability among the cells treated with different concentrations (10 μg/mL, 25 μg/mL, 50 μg/mL, 100 μg/mL, 200 μg/mL, 500 μg/mL and 1000 μg/mL) of β-glu6 was measured by the MTT assay, and even at a concentration of 1000 μg/mL, β-glu6 had no detrimental effects on macrophage viability (Figure 1A). Previously, we demonstrated that β-glu6 enhanced the maturation of HBsAg-induced spleen DCs [6]. This result indicates that β-glu6 might influence the intracellular signaling pathways that mediate DC maturation. The PI3K-Akt signaling pathway is a central convergence node in a broadly influential signaling network, so we examined the effect of β-glu6 on the phosphorylation of AKT and found that β-glu6 suppressed the phosphorylation of AKT (Thr308) in a dose-dependent manner. At the concentration of 100 μg/mL, β-glu6 significantly inhibited the phosphorylation of AKT (Thr308) (Figure 1B). Therefore, β-glu6 was used at a concentration of 100 μg/mL in subsequent in vitro studies.

### β-glu6 (G) modulates the PI3K/Akt signaling pathway

**Figure 2:**
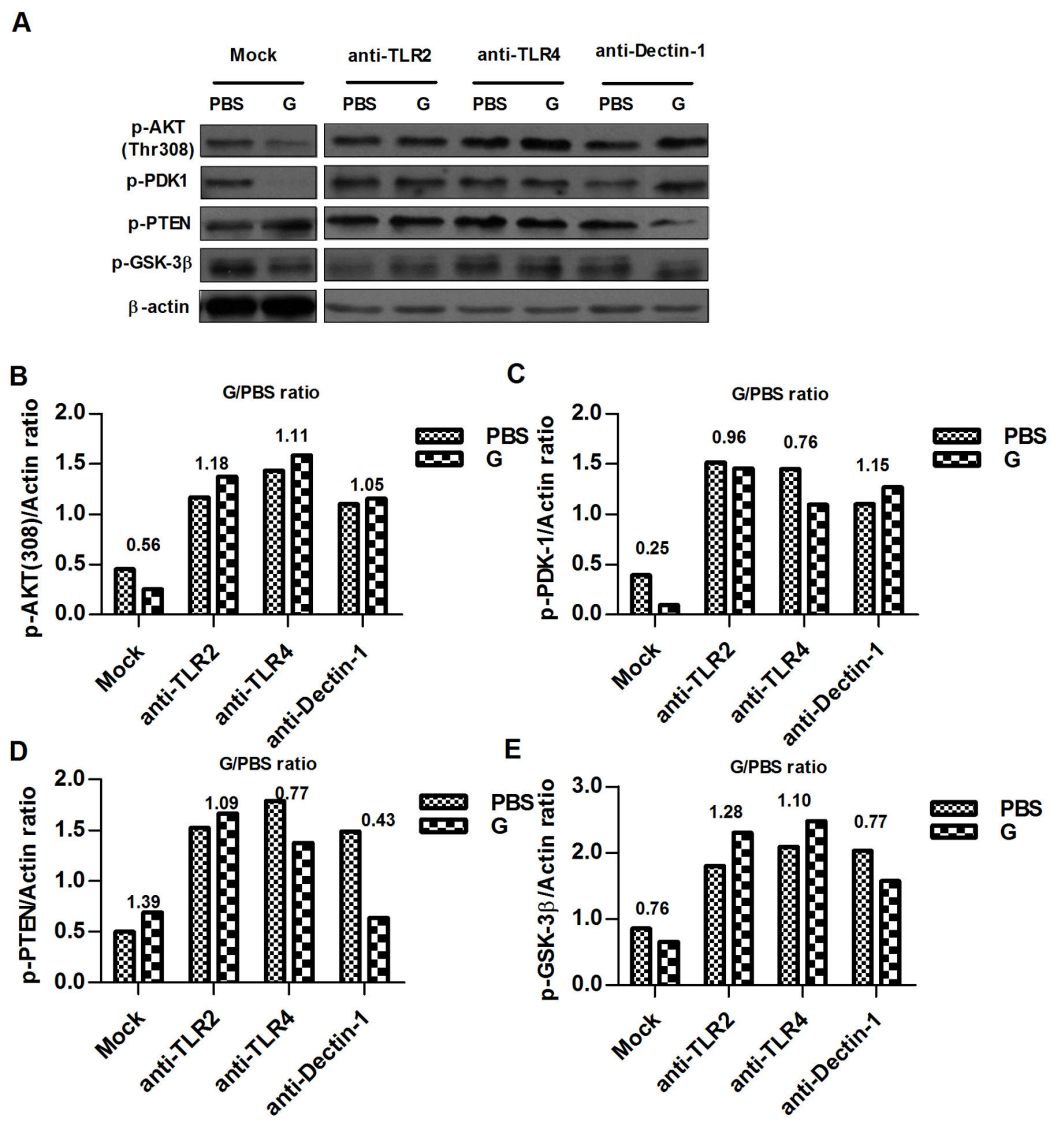
β-glu6 (G) modulates the PI3K/Akt signaling pathway. Macrophages were incubated with β-glu6 (100 μg/mL) alone for 2 h or pre-treated with anti-TLR2, anti-TLR4 or anti-Dectin-1 antibodies for 1 h. The Akt (Thr308), PTEN, PDK-1, and GSK-3β phosphorylation were detected by western blot assay, and beta-actin was used as a loading control (A). The band intensities were analyzed by Quantity One software (B, C, D and E). The numbers above the bars stand for the G/PBS ratio. Data shown represent one of three independent experiments.

Because β-glu6 suppressed the phosphorylation of Akt (Thr308) in a dose-dependent manner in murine macrophages (Figure 1B), we further examined the phosphorylation status of three other proteins, PDK1, PTEN and GSK-3β, in the PI3K/Akt signaling pathway. After 2 h of exposure to β-glu6 (100 μg/mL), the phosphorylation of GSK-3β at Ser9 was diminished. Meanwhile, the phosphorylation of PDK1, a serine/threonine kinase that phosphorylates Akt at Thr-308, was suppressed after β-glu6 treatment. In addition, the phosphorylation level of PTEN, which functions as a tumor suppressor by negatively regulating the Akt signaling pathway, was increased as shown in Figure 2.

We further investigated the potential cell surface molecules involved in the β-glu6-mediated Akt signaling pathway by incubating the murine macrophages with anti-TLR2, anti-TLR4 and anti-Dectin-1 antibodies. The β-glu6-induced reduction in Akt (Thr-308) and PDK1 phosphorylation was reversed. Incubation with anti-TLR4 and anti-Dectin-1 significantly reversed the β-glu6-induced increase of PTEN phosphorylation, while incubation with anti-TLR2 and anti-TLR4 reversed the β-glu6-induced reduction of GSK-3β phosphorylation (Figure 2).

### β-glu6 activates ERK1/2 pathway through TLR2 and Dectin-1

**Figure 3:**
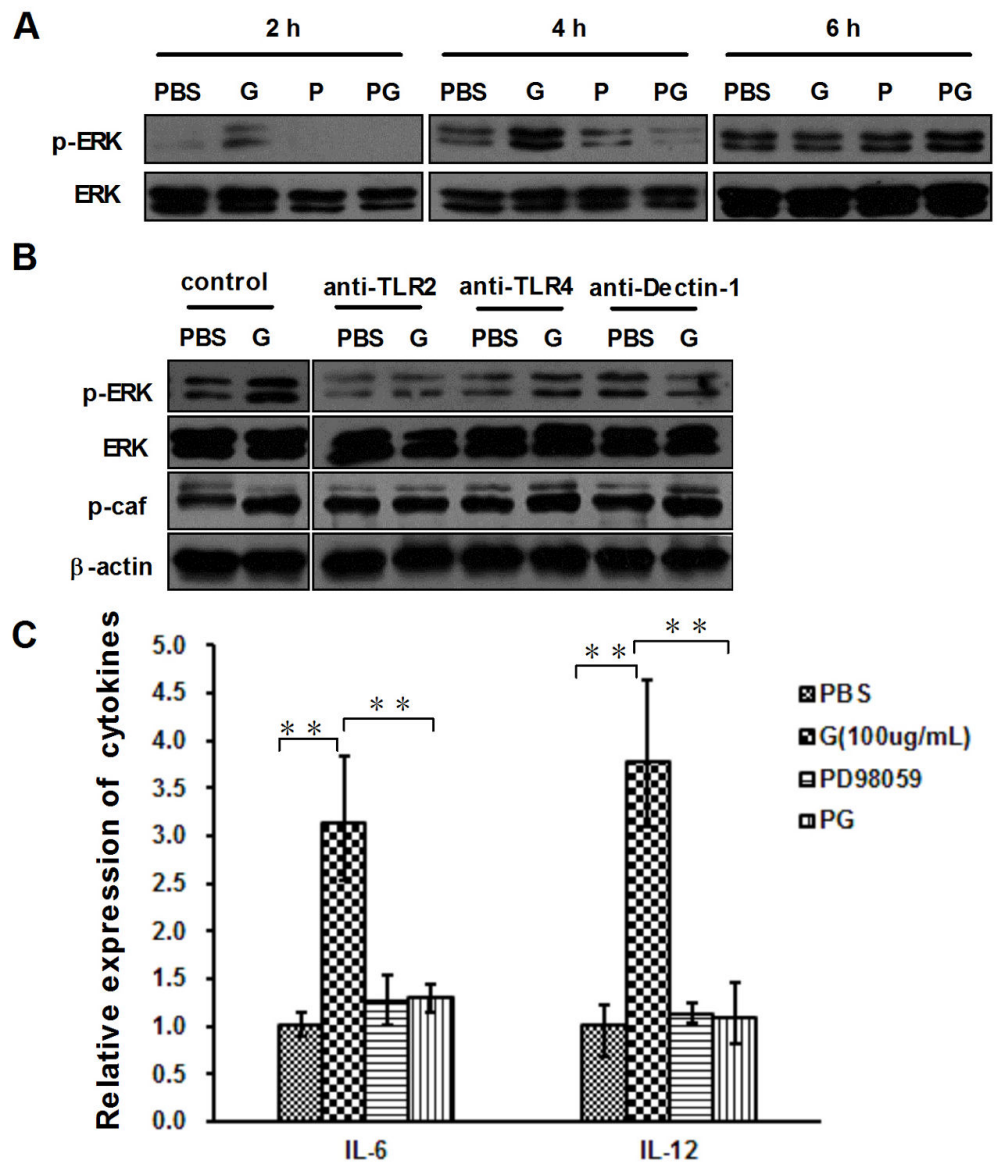
β-glu6 (G) modulates ERK 1/2 phosphorylation and cytokine mRNA expression via TLR2 and Dectin-1. Macrophages were pretreated with PD98059 (P, 50 μM) (A, C) or anti-TLR2, anti-TLR4 or anti-Dectin-1 antibodies (B) for 1 h; the cells were incubated with β-glu6 (G, 100 μg/mL). The levels of ERK 1/2 (both total and phosphorylated), phosphorylated c-Raf and beta-actin were detected by western blot assay. Real-time PCR was used to examine IL-6 and IL-12 mRNA expression levels in macrophages incubated with β-glu6 (G, 100 μg/mL) for 4 h (C). PG: pretreated with PD98059 (P, 50 μM) and incubated with β-glu6 (G, 100 μg/mL). Data are representative of three experiments. ** P<0.01.

ERK1/2 signaling pathway has been reported to regulate cytokine expression and the proliferation of activated macrophages [19]. We examined the phosphorylation status of ERK1/2 and c-Raf, which is a MAP kinase kinase kinase (MAP3K), in β-glu6-treated murine macrophages by Western blot analysis. Treatment with β-glu6 for 2 h activated ERK1/2 and c-Raf in murine macrophages as shown in Figure 3(A, B). We next studied the effect of anti-TLR2, anti-TLR4 and anti-Dectin-1 antibodies (10 μg/mL) on β-glu6-induced ERK1/2 and c-Raf activation. The anti-TLR2 and anti-Dectin-1 incubation reversed the β-glu6-induced increase of ERK1/2 phosphorylation, but anti-TLR4 had no such effect. The increased phosphorylation of c-Raf was markedly reversed by anti-TLR2 incubation but not anti-Dectin-1 incubation (Figure 3B). Moreover, mouse IgG incubation did not change the phosphorylation of ERK and c-Raf (Figure S1). 

The activation of macrophage ERK1/2 by β-glu6 was confirmed using an ERK1/2 inhibitor, PD98059 (Figure 3A). Murine macrophages were pretreated with PD98059 (P, 50 μM) for 1 h, and the mRNA levels of IL-6 and IL-12 were detected at 4 h after β-glu6 was added. The β-glu6-induced IL-12 and IL-6 expression was significantly blocked by pretreatment with PD098059 (Figure 3C).

### β-glu6 induces an M1 like phenotype in murine macrophages

Next, we explored whether β-glu6 is a potential inducer of cytokines and chemokines. Comparative quantiﬁcation of TNF-α, IL-12, IL-1β, IL-6, IL-10, IL-1ra, IL-4 and IFN-γ mRNA expression in β-glu6-treated murine macrophages was performed by real-time RT-PCR. In the murine macrophages treated with β-glu6, the mRNA levels of M1-related inflammatory cytokines TNF-α (24 h), IL-12 (4, 24 h), IL-6 (4 h) and IL-1β (24 h) were greatly increased compared with the control groups; however, the mRNA levels of IL-10 were decreased to 21% (4 h) and 48% (24 h) of that of the control cells. (Table 2). No significant change (>2 fold) was observed in IL-4 expression, while the expression of IFN-γ decreased at 4h and slightly increased at 24h (Table S2).

**Table 2:** β-glu6 modulate M1/M2 cytokines mRNA expression in macrophages.

Cytokines		Mean (range) value of cytokines mRNA expression ratio (β-glu6 treated vs. control)	
		4h	24h
	TNF-α	0.82	3.03*
		(0.78-0.87)	(2.65-3.41)
	IL-12	2.56*	51.50*
M1		(2.45-2.67)	(48.14-54.86)
	IL-1β	1.13	5.87*
		(1.07-1.20)	(5.39-6.35)
	IL-6	2.72*	0.86
		(2.66-2.79)	(0.72-1.00)
	IL-10	0.21**	0.48**
M2		(0.15-0.27)	(0.39-0.57)
	IL-1Ra	1.17	0.99
		(1.13-1.23)	)0.82-1.16)

Data in bold are those with >2-fold difference. * up-regulation (>2-fold increase vs. control), ** down-regulation (>2-fold decrease vs. control). Data are presented as 2^- ΔΔCtmean^ [2^-(ΔΔCt+SD)^, 2^-^(^ΔΔCt–SD)^], ΔΔCt= (Ct_Gene_-Ct _GAPDH_) treatment-(Ct_Gene_-Ct_GAPDH_) control.

**Figure 4:**
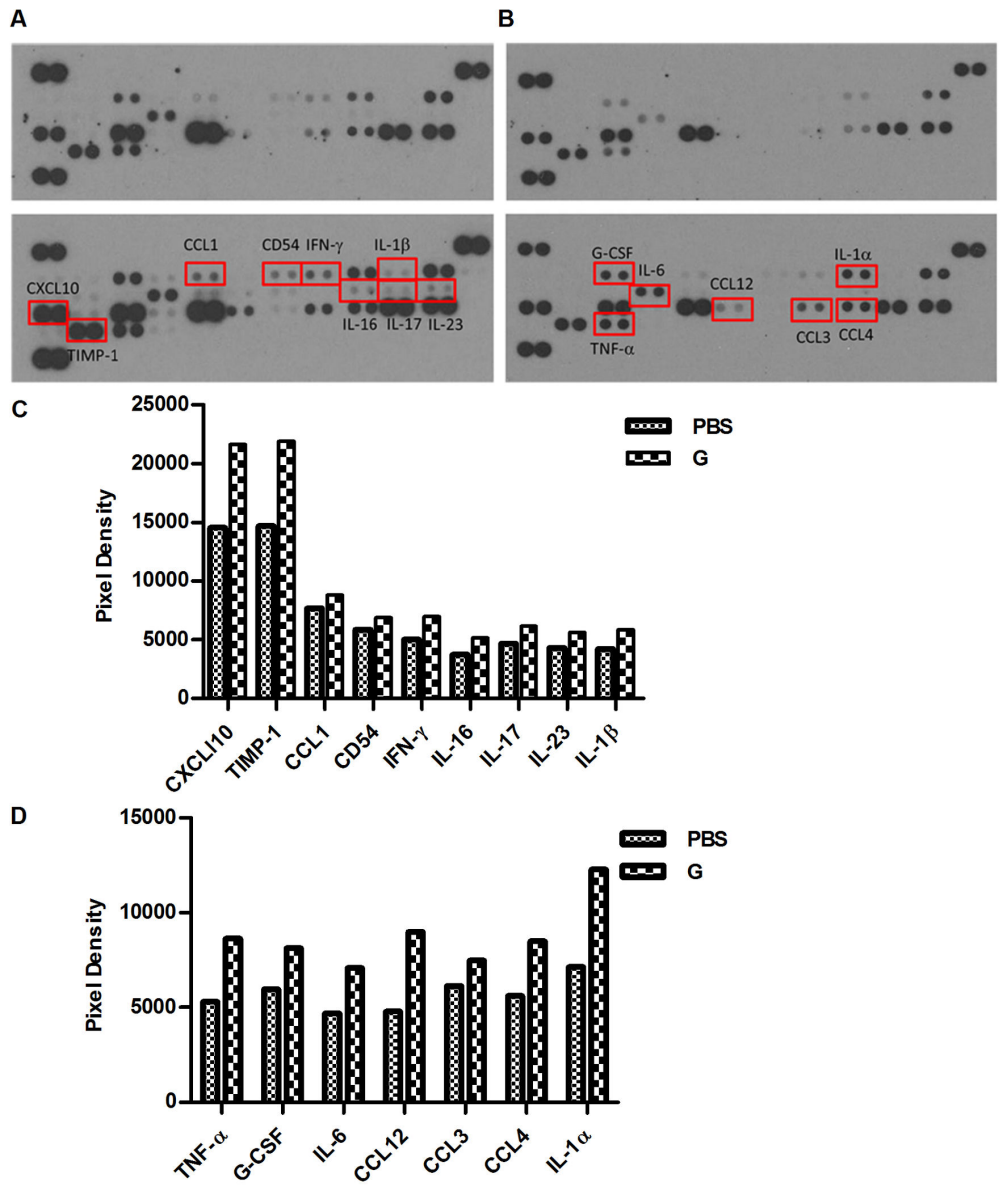
β-glu6 modulates the production of cytokines and chemokines in murine macrophages. Macrophages were incubated with β-glu6 (100 μg/mL) (lower panels in A and B) or PBS (upper panels in A and B) in medium for 24 h, and the cell culture supernatants were collected. The mouse cytokine array Panel A was used to detect the production of cytokines and chemokines according to the manufacturer’s instructions. The densitometric analysis of spot intensity at 5 min (A) and 1 min (B) after exposure was analyzed by Quantity One software (C, D). Data represent one of three representative experiments.

The differential transcription of M1/ M2 cytokine genes in murine macrophages was confirmed by a mouse cytokine array. After 24 h of β-glu6 treatment, the secretion of IL-6, TNF-α, IL-16, IL-17, IL-23, IL-1α, IL-1β and IFN-γ in murine macrophages was markedly enhanced. In addition, the production of CCL1, CCL3, CCL4, CCL12, CXCL10, TIMP-1 and G-CSF was greatly increased (Figure 4).

### β-glu6 exerts immunomodulatory activity in human PBMCs

**Figure 5:**
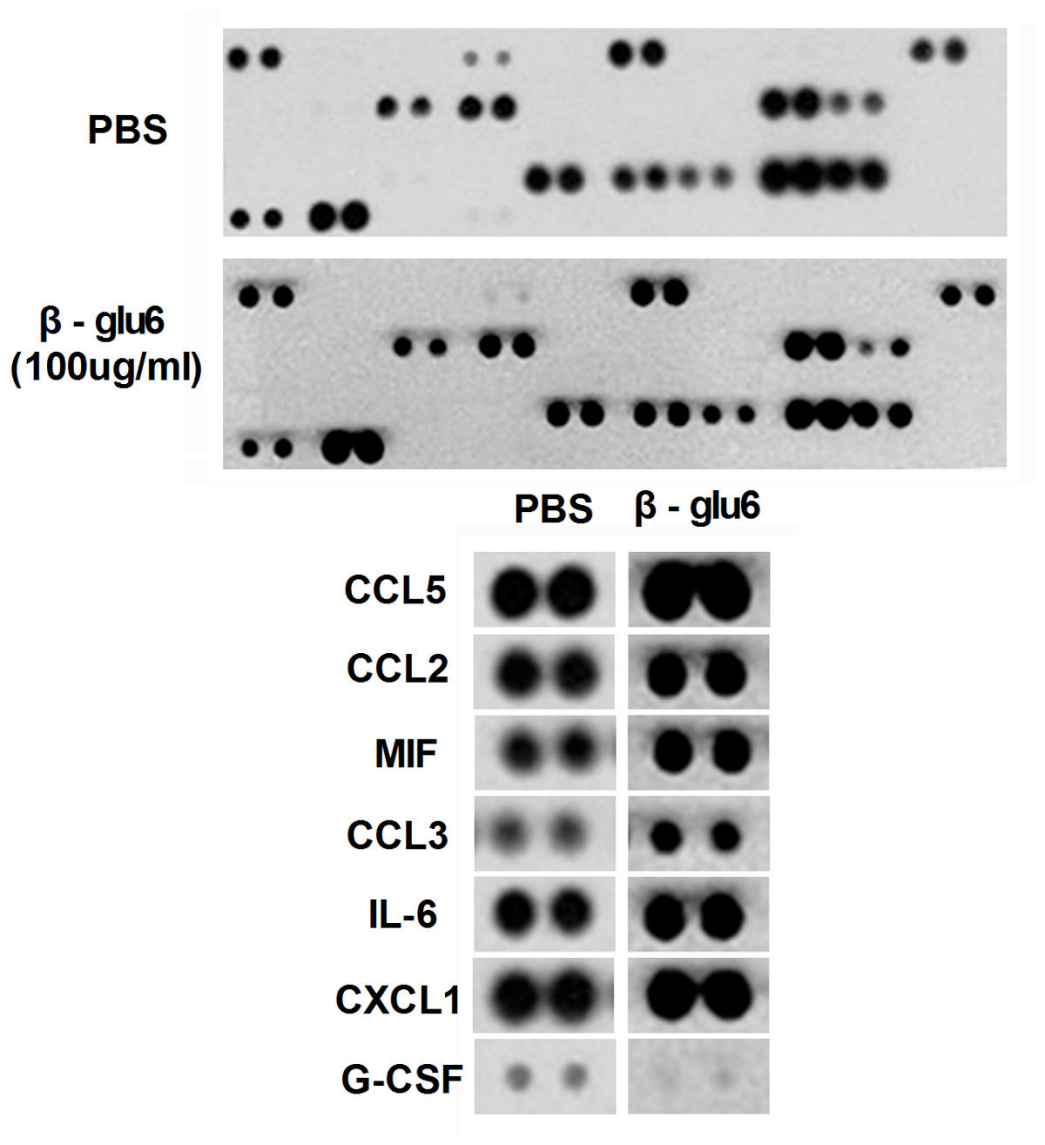
β-glu6 regulates the production of cytokines and chemokines in human PBMCs. Human PBMCs were incubated with β-glu6 (100 μg/mL) (lower panels) or PBS (upper panels) in medium for 24 h, and the cell culture supernatants were collected. The status of the production of cytokines and chemokines was detected by Human Cytokine Array Panel A according to the manufacturer’s instructions. The images below the panels are enlarged dots from Human Cytokine Array Panel A.

 In order to verify the immunomodulatory effects of β-glu6 on human immune cells, we examined the cytokine and chemokine profiles in β-glu6-stimulated human PBMCs. After treated with β-glu6 (100ug/mL) for 24h, the secretion of IL-6, CCL2, CCL3, CCL5, CXCL1 and MIF was enhanced but the secretion of G-CSF was decreased in human PBMCs (Figure 5).

## Discussion

The use of *Lentinus Edodes, Ganoderma lucidum*, and so forth, has long been described in the Compendium of Materia Medica. Polysaccharides and oligosaccharides extracted from *Lentinus edodes* have been used to prevent and treat diseases in traditional Chinese medicine, which has been recorded in the Chinese Pharmacopoeia [20]. Both beta-glucans purified from fungi and synthetic β-glucans have shown immunomodulation activities as well as anti-tumor and anti-microbial activities [21-29]. It has been reported that the extracts of *Lentinus Edodes* fruiting body consists of at least six monosaccharide units [30], therefore, it is difficult to obtain lentinan with high purity. The synthesized glucohexaose with the basic structure of lentinan (β-glu6) maintains the activity of lentinan [2] and its purity is more than 98% [6]. High-yield chemical synthesis of β-glu6 may greatly promote its clinical applications. Meanwhile, it is more convenient to clarify the immunomodulatory mechanisms of a single component than natural exacts.

 Beta-glucans obtained from various sources or prepared under different conditions may exert different regulatory effects. Beta-glucans from aqueus extract of a medicinal mushroom Phellinus linteus (PL), which contains mixed α-and β-linkages, and α (1→6)-branched type (1→3)-glycan, has been reported to inhibit proliferation and colony formation of highly invasive human breast cancer cells by suppressing AKT signaling pathways [31]. However, the polysaccharide component with a branched (1-->3)-beta-D-glucan moiety from *G. lucidum* induced anti-apoptotic effects on neutrophils through activation of Akt signaling pathway [32]. In the present study, the β-glu6, a β-(1→6)-branched β-(1→3) glucohexaose analog contains an α-(1→3)-linked bond, diminished the phosphorylation of Akt at Thr-308, GSK-3β ( a AKt kinase substrate) at Ser9 and the phosphorylation of PDK1( which transduces signals from PI3K to Akt), but activated the phosphorylation of PTEN (which opposes PI3K function, leading to inactivation of AKT), which are consistent with the effects of Phellinus linteus extracts on human breast cancer cells [31].

 We have found that β-glu6 activated ERK1/2 phosphorylation via the TLR2 and Dectin-1 receptors. However, the phosphorylation of c-Raf, a MAP kinase kinase kinase (MAP3K), was decreased in anti-TLR2-treated cells but not in anti- Dectin-1-treated cells (Figure 3B), suggesting that the TLR2 and Dectin-1 signaling pathways may cross at the ERK but not the MAP3K (c-Raf) level. Dectin-1 has been reported to synergize with TLR2 and TLR4 for cytokine production in human primary macrophages [33]. In this study, Dectin-1 synergized with TLR2 and TLR4 in AKT phosphorylation and other downstream events after β-glu6 treatment. However, reports on the involvement of TLR4 in the immunomodulatory effects of β-glucans are controversial. One study showed that TLR4 recognized the *C. albicans* cell wall, which is mainly composed of β-1, 3-glucans, but not all strains of *C. albicans* can be recognized by TLR4 [34]. Further studies are warranted to investigate the interaction between β-glu6 and TLR4.

Previous studies showed that lentinan elicited peritoneal macrophages with increased secretion of IL-12 in vitro and reduced IL-10 in vivo [35] and enhanced the production of IL-1β and TNF-α in peripheral monocytes from the patients with gastric carcinoma [36]. Lentinan induced the enhancement of IL-12, IFN-γ and NO production in spleen cells of malaria infected mice [37] as well as the TNF-α and IL-12 production in bone marrow macrophages and dendritic cells after Listeria monocytogenes infection [38]. In the present study, β-glu6 up-regulated the mRNA levels of TNF-α (24 h), IL-12 (4, 24 h), IL-6 (4 h) and IL-1β (24 h) and down-regulated the mRNA levels of IL-10 (4, 24 h) in murine peritoneal macrophages. It showed that lentinan and β-glu6 have similar effects on cytokine production in macrophages, toward an M1-like phenotype. However, when administrated with different types of antigens, β-glu6 facilitated distinct types of T cell responses: β-glu6 enhanced the virus-specific CTL and Th1 responses induced by a DNA vaccine in C57BL/6 mice [7], while initiating a shift toward Th2-biased immune response in HBsAg immunized BALB/c mice [6]. The dual modulatory role of β-glu6 in immune responses depends on the presence or absence of antigens and the type, conformation and the dose of the antigens [39].

In our previous research, we found a Th2 bias responses in β-glu6 plus HBsAg treated mice, in which β-glu6 enhanced HBsAg specific immune response as an adjuvant. After stimulation with HBsAg in vitro, the number of HBsAg-specific IL-4-producing splenocytes from β-glu6 treated mice was 3 fold higher than that of mice immunized with HBsAg alone, while there were no significant differences in the number of IFN-γ-producing splenocytes between the two groups [6]. However, in the present study, murine peritoneal macrophages were treated with β-glu6 alone and there was little change in IL-4 mRNA expression, indicating that as an immune modulator, β-glu6 has little effect on IL-4 expression in murine peritoneal macrophages in vitro. 

 Previous studies have shown that PI3K-Akt pathway negatively regulated LPS-induced TNF-α, IL-6, and tissue factor gene expression in monocytes/macrophages [40]. Beta-glucan activates microglia through influence AKT signaling pathway without inducing cytokine production [41]. However, in the present study, we only observed the correlation of Ras-Raf-MEK-ERK pathway with cytokine secretion. Further studies will be needed to clarify the role of PI3K-Akt signaling pathway in β-glu6 induced production of cytokines. 

 The present study suggests that the β-glu6-induced secretion of M1 cytokines and chemokines in C57BL/6 peritoneal macrophages is mediated by the Ras-Raf-MEK-ERK signaling pathway via TLR2 and Dectin-1. Moreover, β-glu6 may alert the activation status of PI3K-Akt pathway via TLR2, TLR4 and Dectin-1 receptors. Beta-glu6 has been shown to exert immunoregulatory effects on HBsAg vaccination in BALB/c mice in vivo [6] as well as the immunoregulatory activities in human PBMCs, which suggest its potential use as an adjuvant for vaccines development or as an immunostimulant in anti-infective therapy. Another issue of concern is the dose of β-glu6 used in this study. To address this problem, we have begun to modify the structure of β-glu6 to improve its immunomodulatory properties and thereby reducing its dosage in future researches or clinic application. 

## Supporting Information

Figure S1 **Mouse IgG incubation does not change the phosphorylation of ERK and c-Raf.** Macrophages were pretreated with or without mouse IgG (10μg/mL) for 1 h; the cells were incubated with β-glu6 (G, 100 μg/mL) for 2h. The levels of ERK 1/2 (both total and phosphorylated), phosphorylated c-Raf and beta-actin were detected by Western blot assay.
(TIF)

Table S1 **Detection of Endotoxin in β-glu6 with Limulus Amebocyte Lysate assay.** ^*^Three concentrations (0.1, 0.33 and 1mg/mL) of β-glu6 with or without 0.5EU/mL LPS were added into the appropriate microplate well and detected with (LAL) QCl-1000 kit, measured in Endotoxin Units per milliliter (EU/mL).
^**^ LPS concentration was 0.5 EU/mL.
^***^N.D.: Not Detectable.
(DOC)

Table S2 **Beta-glu6 modulates cytokine gene expression in murine macrophages.** Data in bold are those with >2-fold difference. * Down-regulation (>2-fold decrease vs. control). Data are presented as 2^-ΔΔCtmean^ [2^-(ΔΔCt+SD)^, 2^-^(^ΔΔCt–SD)^], ΔΔCt= (Ct_Gene_-Ct _GAPDH_) treatment-(Ct_Gene_-Ct_GAPDH_) control.
(DOC)
