# Supplementary figures and images for: Immunomodulatory Activity of a Novel, Synthetic Beta-glucan (β-glu6) in Murine Macrophages and Human Peripheral Blood Mononuclear Cells
Source: PLoS One. 2013 Nov 6;8(11):e80399. doi: 10.1371/journal.pone.0080399 (PMC3819285; doi:10.1371/journal.pone.0080399)

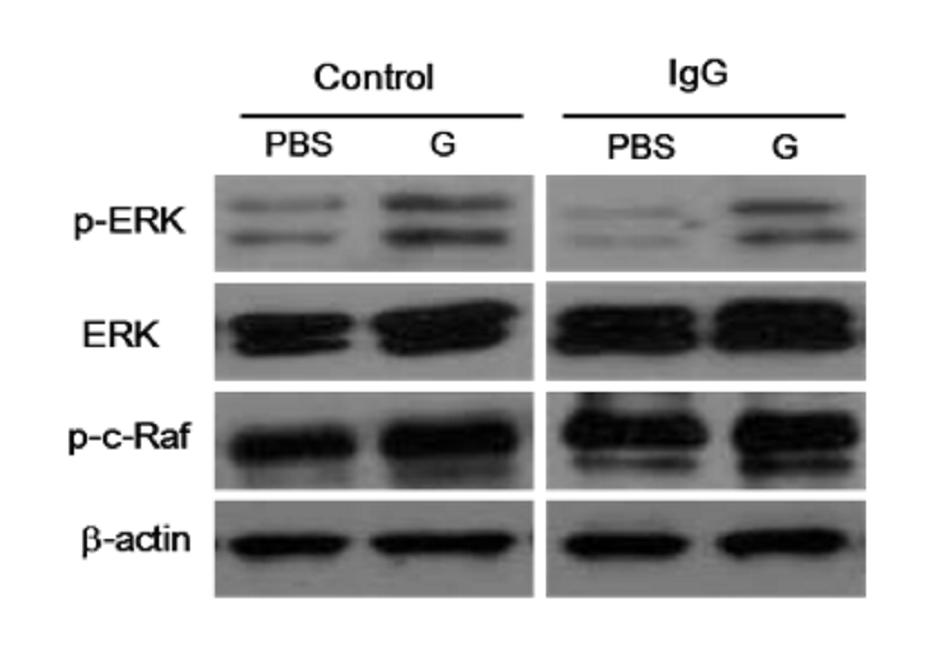

Supplement: Figure S1 — Mouse IgG incubation does not change the phosphorylation of ERK and c-Raf. Macrophages were pretreated with or without mouse IgG (10μg/mL) for 1 h; the cells were incubated with β-glu6 (G, 100 μg/mL) for 2h. The levels of ERK 1/2 (both total and phosphorylated), phosphorylated c-Raf and beta-actin were detected by Western blot assay. (TIF) [file pone.0080399.s001.tif]
