# Supplementary material for: Immunomodulatory Activity of a Novel, Synthetic Beta-glucan (β-glu6) in Murine Macrophages and Human Peripheral Blood Mononuclear Cells
Source: PLoS One. 2013 Nov 6;8(11):e80399. doi: 10.1371/journal.pone.0080399 (PMC3819285; doi:10.1371/journal.pone.0080399)
Supplement: Table S1 — Detection of Endotoxin in β-glu6 with Limulus Amebocyte Lysate assay. *Three concentrations (0.1, 0.33 and 1mg/mL) of β-glu6 with or without 0.5EU/mL LPS were added into the appropriate microplate well and detected with (LAL) QCl-1000 kit, measured in Endotoxin Units per milliliter (EU/mL). ** LPS concentration was 0.5 EU/mL. ***N.D.: Not Detectable. (DOC) [file pone.0080399.s002.doc]

**Supplementary Table1. Detection of Endotoxin in β-glu6 with Limulus** Amebocyte Lysate assay.

| Group | OD405 | EU/mL^*^ |
| --- | --- | --- |
| β-glu6 (0.1mg/mL) | 0.118±0.001 | *N.D.* ^***^ |
| β-glu6 (0.33mg/mL) | 0.121±0.004 | *N.D.* |
| β-glu6 (1mg/mL) | 0.120±0.003 | *N.D.* |
| β-glu6 (0.1mg/mL)+ LPS^**^ | 0.564±0.003 | 0.5 EU |
| β-glu6 (0.33mg/mL)+ LPS^**^ | 0.553±0.002 | 0.5 EU |
| β-glu6 (1mg/mL)+ LPS^**^ | 0.560±0.004 | 0.5 EU |
| LPS^**^ | 0.568±0.003 | 0.5 EU |
| LAL water | 0.078±0.043 | *N.D.* |
| PBS | 0.075±0.037 | *N.D.* |

^*^Three concentrations (0.1, 0.33 and 1mg/mL) of β-glu6 with or without 0.5EU/mL LPS were added into the appropriate microplate well and detected with (LAL) QCl-1000 kit, measured in Endotoxin Units per milliliter (EU/mL).

^**^ LPS concentration was 0.5 EU/mL.

^***^N.D.: Not Detectable.
