# Supplementary material for: Immunomodulatory Activity of a Novel, Synthetic Beta-glucan (β-glu6) in Murine Macrophages and Human Peripheral Blood Mononuclear Cells
Source: PLoS One. 2013 Nov 6;8(11):e80399. doi: 10.1371/journal.pone.0080399 (PMC3819285; doi:10.1371/journal.pone.0080399)
Supplement: Table S2 — Beta-glu6 modulates cytokine gene expression in murine macrophages. Data in bold are those with >2-fold difference. * Down-regulation (>2-fold decrease vs. control). Data are presented as 2-ΔΔCtmean [2-(ΔΔCt+SD), 2-(ΔΔCt–SD)], ΔΔCt= (CtGene-Ct GAPDH) treatment-(CtGene-CtGAPDH) control. (DOC) [file pone.0080399.s003.doc]

**Supplementary Table 2. Beta-glu6 modulates cytokine gene expression in murine macrophages**

| Cytokines | Mean (range) value of cytokines mRNA expression ratio (β-glu6 treated vs. PBS control) | |
| --- | --- | --- |
|  | 4h | 24h |
| IL-4 | 1.01 | 1.43 |
|  | (0.92-1.10) | (1.27-1.59) |
| IFN-γ | **0.44*** | 1.71 |
|  | (0.37-0.51) | (1.52-1.90) |

Data in bold are those with >2-fold difference. ***** Down-regulation (>2-fold decrease vs. control). Data are presented as 2^-ΔΔCtmean^ [2^-(ΔΔCt+SD)^, 2^-(ΔΔCt–SD)^], ΔΔCt= (Ct_Gene_–Ct _GAPDH_) treatment–(Ct_Gene_–Ct_GAPDH_) control.
